# A low-carbohydrate high-fat diet decreases lean mass and impairs cardiac function in pair-fed female C57BL/6J mice

**DOI:** 10.1186/s12986-016-0132-8

**Published:** 2016-11-15

**Authors:** Jessica Nilsson, Madelene Ericsson, Masoumeh Motamedi Joibari, Fredrick Anderson, Leif Carlsson, Stefan K. Nilsson, Anna Sjödin, Jonas Burén

**Affiliations:** 1 Department of Medical Biosciences, Physiological Chemistry, Umeå University, SE-901 87 Umeå, Sweden; 2 Department of Public Health and Clinical Medicine, Medicine, Umeå University, Umeå, Sweden; 3 Umeå Centre for Molecular Medicine, Umeå University, Umeå, Sweden; 4 Department of Food and Nutrition, Umeå University, Umeå, Sweden

**Keywords:** Low-carbohydrate diet, Heart, Mouse

## Abstract

**Background:**

Excess body fat is a major health issue and a risk factor for the development of numerous chronic diseases. Low-carbohydrate diets like the Atkins Diet are popular for rapid weight loss, but the long-term consequences remain the subject of debate. The Scandinavian low-carbohydrate high-fat (LCHF) diet, which has been popular in Scandinavian countries for about a decade, has very low carbohydrate content (~5 E %) but is rich in fat and includes a high proportion of saturated fatty acids. Here we investigated the metabolic and physiological consequences of a diet with a macronutrient composition similar to the Scandinavian LCHF diet and its effects on the organs, tissues, and metabolism of weight stable mice.

**Methods:**

Female C57BL/6J mice were iso-energetically pair-fed for 4 weeks with standard chow or a LCHF diet. We measured body composition using echo MRI and the aerobic capacity before and after 2 and 4 weeks on diet. Cardiac function was assessed by echocardiography before and after 4 weeks on diet. The metabolic rate was measured by indirect calorimetry the fourth week of the diet. Mice were sacrificed after 4 weeks and the organ weight, triglyceride levels, and blood chemistry were analyzed, and the expression of key ketogenic, metabolic, hormonal, and inflammation genes were measured in the heart, liver, and adipose tissue depots of the mice using real-time PCR.

**Results:**

The increase in body weight of mice fed a LCHF diet was similar to that in controls. However, while control mice maintained their body composition throughout the study, LCHF mice gained fat mass at the expense of lean mass after 2 weeks. The LCHF diet increased cardiac triglyceride content, impaired cardiac function, and reduced aerobic capacity. It also induced pronounced alterations in gene expression and substrate metabolism, indicating a unique metabolic state.

**Conclusions:**

Pair-fed mice eating LCHF increased their percentage of body fat at the expense of lean mass already after 2 weeks, and after 4 weeks the function of the heart deteriorated. These findings highlight the urgent need to investigate the effects of a LCHF diet on health parameters in humans.

## Background

The prevalence of obesity has increased in the last few decades, as have the metabolic syndromes and cardiac health problems that are associated with obesity [1]. This is most likely due to both behavioral and environmental factors, such as changes in dietary habits and activity levels, rather than to genetic factors. During the last ten years, a variety of low-carbohydrate high-fat diets have become more popular, like the Atkins diet and the Scandinavian low-carbohydrate high-fat diet (LCHF). This is probably because such diets can help people lose weight rapidly. The Swedish Council on Health Technology Assessment recently performed a systematic knowledge survey on the dietary treatment of obesity [2]. The authors concluded that in the short-term, i.e., over a 6-month period, providing advice about strict or moderately low-carbohydrate diets is more effective for weight loss than providing advice about low-fat diets.

The present study aimed to investigate the metabolic and physiological consequences of a diet that has a macronutrient composition similar to the Scandinavian LCHF diet [75 energy proportion (E %) fat, 20 E % protein, 5 E % carbohydrates]. The Scandinavian LCHF diet has a high fat content, and the fat comes mostly from dairy products and meat, both of which have high levels of saturated fatty acids. This diet also encourages the intake of medium-chain triglycerides. It is currently unknown whether there is a causal link between high fat intake and an increased risk of diseases such as type 2 diabetes and cardiovascular disease [2].

The C57BL/6J mouse model is widely used for dietary studies because they are susceptible to diet-induced obesity and type 2 diabetes. A standard high-fat diet with >45 E % fat, results in weight gain and metabolic syndrome in C57BL/6J mice [3]. Notably, a diet that is extremely high in fat can induce a state of dietary ketosis. The parameters that induce ketosis in mice are not yet well defined. Several studies have proposed that a high ratio of fat to carbohydrate, usually >80 E % fat and <5 E % carbohydrates, will induce ketosis. In contrast, Bielohuby et al. stated that it is the fat to protein ratio rather than the absence of carbohydrates per se that is important for the development of dietary ketosis [4]. While animals fed a high-fat diet often gain weight due to overconsumption of calories [3], ketogenic diets are often very low in protein and may cause starvation followed by weight loss [3, 5, 6]. Notably, the most commonly used ketogenic diet in mice contains 95 E % fat, 5 E % protein, and 0 E % carbohydrates, which does not resemble any human diet. Moreover, previous studies have not focused on weight stable animals or on diets that are similar to the Scandinavian LCHF diet. Therefore, in the present study, we aimed to investigate the metabolic and physiological consequences of a diet that has a macronutrient composition similar to that of the Scandinavian LCHF diet and, in particular, to investigate the effects of this diet on organs, tissues, and metabolism in weight stable mice. Since there are gender differences in the metabolic response to high fat diets in rodents, we chose to study female mice that are more resistant to diet-induced metabolic changes [7, 8].

## Methods

### Animals

Fourteen 9-week-old wild-type female C57BL/6J mice were purchased from Charles River Laboratories (Germany) and housed at 21 ± 1 °C with a 12:12 h dark-light cycle (dark period 18.00–06.00) and 45–50 % humidity. The mice were acclimatized, housed individually, and weight-matched for 4 weeks before the diet switch. During the acclimatization period, all mice had *ad libitum* access to water and to standard rodent chow (801730; Special Diets Services, Essex, UK) consisting of 9 E % fat, 22 E % protein, and 69 E % carbohydrates.

### Experimental design

Once acclimatized, mice had reached an age of 13 weeks and one mouse in each weight matched pair were randomly selected to have either chow diet (control, *n* = 7) or low-carbohydrate high-fat diet (LCHF, *n* = 7). Each control mouse was fed standard rodent chow with continued free access to food, while its weight-matched mate was pair-fed a LCHF diet (D14090501; Research Diets Inc., NJ, USA). In the LCHF diet, 75 % of the calories were from fat (mainly cocoa butter), 20 % of the calories were from protein, and 5 % were from carbohydrates. Each cage had a protective grid cover that prevented food contamination from nearby cages. Leftover food was weighed on a daily basis, and the consumed caloric intake was calculated for each mouse in the control group. Each mouse in the LCHF group was then iso-energetically fed to match the intake of calories of the (control) matched mouse. The mice were weighed every third day and the animals were sacrificed when 4 weeks of diet was completed.

Blood samples were collected from the tail vein after a 6-h fasting period prior to the diet switch and again at the end of the study prior to sacrifice. At the second time point, additional blood was collected by heart puncture during isoflurane anesthesia. All blood samples were collected in EDTA-coated capillary tubes. Blood samples were centrifuged, and plasma was stored at −80 °C until analysis.

Plasma insulin levels were determined using an enzyme-linked immunosorbent assay (Mouse Insulin ELISA kit, Mercodia, Uppsala, Sweden). Non-esterified fatty acids were measured using an in vitro enzymatic calorimetric method (Wako Life Sciences, Inc., CA, USA), and β-hydroxybutyrate was analyzed using the Beta-Hydroxybutyrate Assay Kit (#MAK041; Sigma Aldrich, MO, USA). All assays were performed according to the manufacturer’s instructions, and all samples were run in duplicate. Fasting blood glucose was measured directly from the tail vein using a hand-held blood glucose meter (Accu-Chek Aviva, Roche Diagnostics Scandinavia, Bromma, Sweden) after a 6-h fasting period prior to the diet switch and at the end of the study.

The mice were dissected, and the weights of the heart, liver, kidney, lung, and spleen were determined. The tibia was collected for gravimetric analysis. For gene expression analysis, tissue samples were collected from the heart, liver, vastus lateralis, gastrocnemius, subcutaneous, and perigonadal adipose tissue and placed into RNAlater buffer (Ambion, Life Technologies, CA, USA) before storage at −18 °C. For tissue triglyceride analysis, the samples were kept on ice after collection and then stored at −18 °C. Samples for lipoprotein lipase activity measurements were snap-frozen in liquid nitrogen and stored at −80 °C until analysis.

### Echocardiography

Transthoracic parasternal long-axis high-frequency echocardiography (Vevo 2100; Visual Sonics, Toronto, ON, Canada) was performed 1 week prior to the diet switch and again after the mice were on the control or the LCHF diet for 4 weeks to assess cardiac function using the model MS-550D scan head (VisualSonics). Images were recorded during light isoflurane anesthesia (1.0–1.75 %; Baxter, Sweden) in 100 % O_2_ (800 ml/min). The anesthesia level was adjusted to keep the respiration rate at 80–100 bpm. Left ventricle end-diastolic and end-systolic volumes were measured by tracing the endocardium in B-mode. The left ventricle wall thicknesses and luminal diameter were measured in M-mode. Data ware stored digitally and analyzed offline (Vevo LAB 1.7.0, Visual Sonics, Toronto, ON, Canada).

### Body composition measurements

Total fat and lean mass were analyzed in fed mice prior to the diet switch and again after 2 and 4 weeks using a fully automatic Echo-MRI system (Echo Medical Systems, TX, USA) according to the manufacturers’ instructions [9].

### Indirect calorimetry and aerobic testing

At week 4, the metabolic rate was measured in 4 mice from each diet group during a 24-h period using the Oxymax chamber (Columbus Instruments, OH, USA) to monitor the respiratory exchange ratio (RER) and energy expenditure (kcal/h).

To investigate aerobic capacity during physical performance, a maximal oxygen consumption (VO_2max_) test was performed prior to the diet switch and after 2 and 4 weeks on the diet using a treadmill (inclination 19°) enclosed in a metabolic chamber (Oxymax, Columbus Instruments, OH, USA). The system was calibrated against a reference gas consisting of 20.5 % O_2_ and 0.5 % CO_2_. Prior to the test, the mice had a 5-min warm-up session at 0.09 m/s and then the speed was gradually increased by 0.03 m/s every other minute until the mice could no longer run on the upper half of the treadmill. All data were normalized to the individual mouse’s body weight.

### Triglyceride and cholesterol plasma profile analysis

Lipoprotein profiles were obtained by size-exclusion chromatography using an automated HPLC system (Elite LaChrom, Hitachi, Krefeld, Germany) as described previously [10]. Plasma samples were diluted 1:16 in elution buffer (150 mM NaCl, 10 mM Tris and 0.02 % NaN_3_) and injected into a Superose 6 gel filtration column (GE Healthcare, Uppsala, Sweden). Triglyceride and cholesterol concentrations were measured online using appropriate triglyceride and cholesterol reagents (Roche, Basel, Switzerland). EZChrom Elite (Agilent Technologies, Boeblingen, Germany) software was used for data processing.

### Lipoprotein lipase activity assay

Lipoprotein lipase (LPL) activity was measured in the heart, subcutaneous, and perigonadal white adipose tissue as described previously [11]. Briefly, triplicate samples were incubated with phenylmethylsulfonyl-treated heat-inactivated frozen rat serum (6 % BSA, 0.1 M NaCl, 0.15 M Tris HCl pH 8.5), Millipore water, and an intralipid with incorporated ^3^H-labelled triolein (PerkinElmer, USA). Samples were then incubated at 25 °C for 2 h. The reactions were stopped with iso-propanol:heptane:1 M H_2_SO_4_:Millipore water (40:48:3:1). Samples were centrifuged and transferred to alkali ethanol and cleaned with heptane. Scintillation fluid (Optiphase, PerkinElmer Inc., MA, USA) was added to the samples in alkali ethanol and a liquid scintillation counter (PerkinElmer, Waltham, MA, Wallac, Win Spectral) was used.

### RNA extraction and real-time quantitative PCR

Total RNA was extracted from left ventricular myocardium, liver, subcutaneous and perigonadal white adipose tissue as described by Alvehus et al. [12]. Samples were run in triplicate using single tube TaqMan gene expression assays (Applied Biosystems) or using primers and probes designed for *Angptl4* and *Lpl* [13] and *αSMA* [14]. The relative mRNA expression levels were calculated using the 2^-ΔΔCt^ method. A reference sample and an endogenous control gene were used to normalize the Ct-values. Expression of 4 endogenous control genes, *Gapdh*, *Rpl32, Polr2a*, and *Ppia*, was evaluated in the entire study cohort to determine their stability using the Normfinder algorithm [15]. *Ppia* was the most stable endogenous control for left ventricular myocardium and subcutaneous white adipose tissue; *Rpl32* was the most stable for perigonadal adipose tissue; and a geometric mean of *Ppia* and *Rpl32* was used for the liver.

### Lipid extraction and tissue triglyceride content

Lipids were extracted according to the method of Willecke et al. with minor modifications [16]. Heart and liver tissue was homogenized in PBS (approximately 50 mg tissue in 1 ml PBS) using stainless steel beads for 5 min at 4 °C in a bead beater homogenizer. The homogenate was vortexed with 2 ml chloroform:methanol (2:1) and centrifuged for 10 min at 4 °C at 830 × *g*. The lower organic phase was collected in a new tube and dried for ~72 h. The dried lipids were then dissolved in 500 μl 1 % Triton-X 100 in chloroform and then dried overnight. Finally, the dried lipids were dissolved in 100 μl of Millipore water. Cardiac and hepatic triglyceride content was measured in duplicate using the Triglyceride GPO-PAP method (Roche Diagnostics, IN, USA).

### Liver histology

Sections (4 μm) of formalin-fixed paraffin-embedded liver tissue were stained with hematoxylin-eosin and with Gordon and Sweet’s stain to visualize the reticular fibers and to reveal liver morphology. The sections were analyzed by light microscopy, and images were collected (NIS-Elements AR 3.22, Nikon Instruments Europe B.V).

### Statistics

The data were analyzed using Graphpad Prism (GraphPad Prism 4, La Jolla, CA, USA). A paired student’s *t*-test was performed for statistical comparisons between the two diet groups. When analyzing the Echo-MRI and the indirect calorimetric data, both diet and time points were taken into consideration using two-way ANOVA with Bonferroni post-tests. We considered *p*-values < 0.05 to be significant. All data are presented as mean ± SD.

## Results

Baseline measurements for blood glucose, NEFA, echocardiography and maximum running speed were performed the week after the animals were weight-matched and before the diets were introduced. No differences were found between the two groups of mice, and baseline values are therefore not presented for those parameters.

### Total caloric intake, weight gain, and changes in body composition

**Fig. 1:**
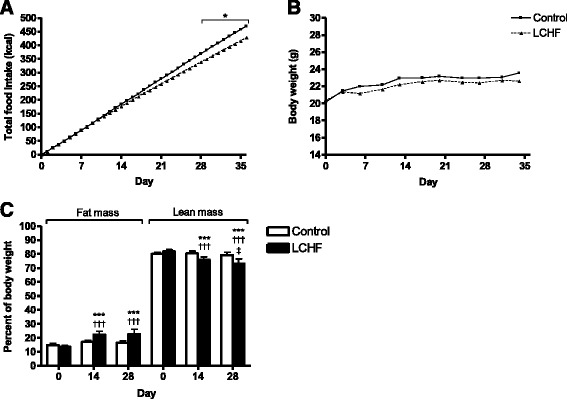
Caloric intake, body weight, and body composition of 14 female C57BL/6J mice fed either regular chow feed (Control) or a low-carbohydrate high-fat (LCHF) diet. Mice fed the LCHF diet maintained their body weight until the end of the study but increased their fat mass. **a** Daily and total caloric intake. During the final week, mice fed the LCHF diet consumed significantly fewer total calories than mice fed the control diet. SD < 8.8 % for both groups at each single data point. **b** Body weight was measured every third day. SD < 6.8 % and < 6.2 % at each single data point for the control and LCHF-fed mice, respectively. **c** Body composition and the percentage of total body weight measured prior to the diet switch (week 0) and after 2 and 4 weeks on the diet. Data are presented as the mean ± SD with *n* = 7 in each group. *p < 0.05, ***p < 0.001 vs. control at the indicated time point; ^†††^ *p* < 0.001 vs. week 0 within each diet group; ^‡^ *p* < 0.05 vs. week 2 within each diet group

During the 4-week study period, the total caloric intake was equal in the two groups until the fourth week, when the mice fed the LCHF diet consumed fewer calories than the controls (Fig. 1a). At the end of the study, the control group and LCHF group had consumed 456 and 417 cal, respectively (*p* < 0.05). Despite the lower total calorie consumption, the total increase in body weight was similar in the two groups (Fig. 1b). Body composition, i.e., the fat and lean mass content, was maintained in the control group, while mice fed the LCHF diet gained fat mass (*p* > 0.001) with a simultaneous reduction in lean mass (*p* < 0.001). These alterations could be seen by the second week (Fig. 1c).

### Plasma parameters and changes in organ weight

At the end of the study, the LCHF diet had not altered the fasting glucose, total triglyceride, insulin, or non-esterified fatty acid plasma levels (Table 1). Mice fed the LCHF diet had lower liver and lung weights than the control mice (Table 2), while the spleen and kidney weights were similar in the two groups. There was a trend for the heart to be lighter in mice fed the LCHF diet, but this did not reach significance because of one strong outlier in the LCHF group. The tibia length was shorter in the LCHF group (*p* < 0.01) (Table 2).

**Table 1:** The effects of a low-carbohydrate high-fat (LCHF) diet on plasma parameters in female C57BL/6J mice

	Control	LCHF
Fasting glucose, mmol/l	7.76 ± 0.48	7.73 ± 1.05
Fasting total TG, mM	0.133 ± 0.068	0.123 ± 0.042
Fasting insulin, ng/ml	0.504 ± 0.039	0.477 ± 0.050
HOMA-IR	4.30 ± 0.24	4.10 ± 1.02
Fasting NEFA, mmol/l	162 ± 56	138 ± 44
β-hydroxybutyrate, mmol/l	0.689 ± 0.239	1.023 ± 0.363

Data are presented as mean ± SD, *n* = 7 in each group

*TG* triglycerides, *NEFA* non-esterified fatty acids

**Table 2:** Organ weight, the organ to body weight ratio, and tibia length in female C57BL/6J mice fed a control diet or a low-carbohydrate high-fat (LCHF) diet

	Control	LCHF
Organ weight, mg		
Heart	98.3 ± 4.3	93.3 ± 8.0
Liver	909 ± 119	753 ± 21**
Lung	135 ± 5	126 ± 3**
Spleen	80.0 ± 4.4	79.3 ± 13.1
Kidney	137 ± 14	131 ± 12
Organ/BW ratio, mg/g		
Heart	4.3 ± 0.3	4.2 ± 0.2
Liver	39.9 ± 4.3	33.9 ± 1.6**
Lung	5.9 ± 0.2	5.7 ± 0.2**
Tibia, mm	17.6 ± 0.2	17.3 ± 0.1**

Data are presented as mean ± SD, *n* = 7 in each group. *BW* body weight. **p < 0.01 was considered significant vs. control diet

### Cardiac function, aerobic capacity, and gene expression analysis

**Fig. 2:**
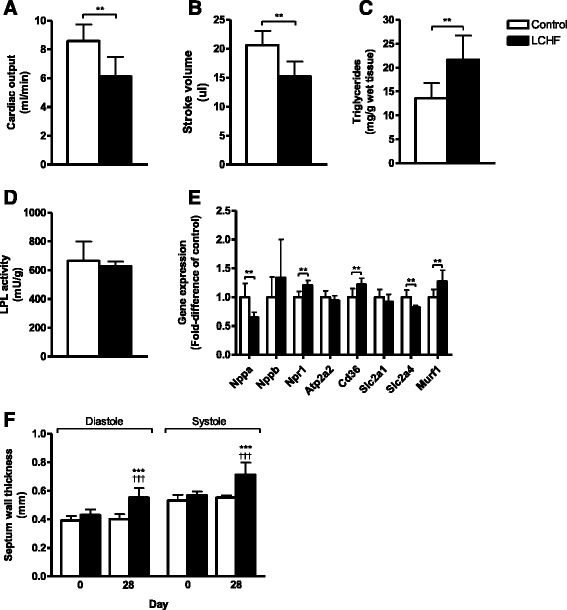
Female C57BL/6J mice fed a low-carbohydrate high-fat (LCHF) diet had impaired cardiac function and early signs of myocardial triglyceride accumulation compared with mice fed regular chow feed (Control). **a** Cardiac output. **b** Stroke volume. **c** Triglyceride content per gram wet cardiac tissue. **d** Lipoprotein lipase (LPL) activity in cardiac tissue. **e** Relative expression of genes involved in the transport of fatty acid and glucose in cardiac tissue after normalization to expression in control mice. **f** Septum wall thickness at diastole and systole at baseline and after 4 weeks on each diet. Data are presented as mean ± SD with *n* = 7 in each group. **p < 0.01 vs. control at the indicated time point; ^†††^ *p* < 0.001 vs. week 0 within each diet group

After 4 weeks on diet, echocardiography demonstrated impaired cardiac function in the LCHF group compared to the control group in terms of reduced stroke volume and cardiac output (*p* < 0.05) (Fig. 2a and b). Contractility, measured as fractional shortening, was also impaired in the LCHF group relative to the control group (*p* < 0.05), and the septum wall showed signs of hypertrophy in both systole and diastole in the LCHF group (*p* < 0.01) (Fig. 2f). Finally, the maximum aerobic capacity was lower in the LCHF group at the end of the study compared to the control group (oxygen uptake capacity 41.05 ± 1.84 vs. 38.58 ± 2.13 ml/kg^0.75^/min, *p* < 0.05; running capacity 0.27 ± 0.04 vs. 0.22 ± 0.05 m/s, *p* = 0.059, for mice fed the control diet and the LCHF diet, respectively).

The cardiac triglyceride content was higher in mice fed the LCHF diet compared to the control mice (*p* < 0.05); however, there was no difference in the LPL activity (Fig. 2c and d). The gene expression analysis focused on genes involved in fatty acid and glucose uptake (Fig. 2e). The atrial natriuretic peptide gene *Nppa* was expressed at significantly lower levels in the LCHF group than in the control group (*p* < 0.01), but the expression of the gene encoding its receptor, natriuretic peptide receptor 1 (*Npr1*), was higher in the LCHF group (*p* < 0.01). Furthermore, expression of the fatty acid translocase *Cd36* gene was higher in the LCHF group (*p* < 0.01), but the expression of the insulin-regulated glucose transporter 4 (*Slc2a4*) was lower (*p* < 0.01) (Fig. 2e). Expression of the muscle ring finger 1 gene, *Murf1*, was higher in the LCHF group compared to the control group (*p* < 0.01), while the expression of the sarco/endoplasmic reticulum Ca^2+^-ATPase, *Atp2a2*, was similar in both groups.

### Adipose tissue

**Fig. 3:**
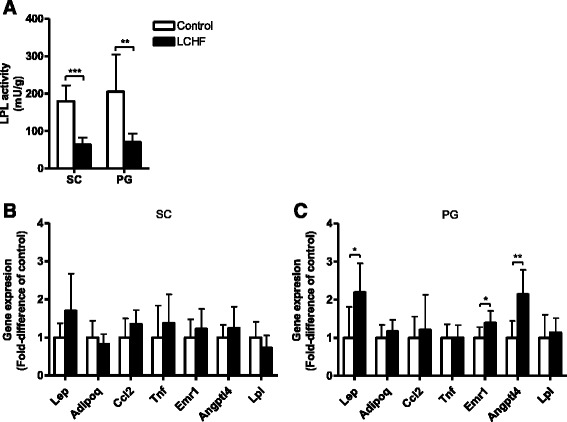
Female C57BL/6J mice fed a low-carbohydrate high-fat (LCHF) diet showed lower lipoprotein lipase (LPL) activity in the subcutaneous (SC) and perigonadal (PG) white adipose tissue than mice fed a regular chow feed diet (Control). **a** LPL activity in SC and PG white adipose tissue. **b** Relative gene expression in SC white adipose tissue normalized to that in control mice. **c** Relative gene expression in PG white adipose tissue normalized to that in control mice. Data are presented as mean ± SD with *n* = 7 in each group. *p < 0.05, **p < 0.01, ***p < 0.001 vs. control

The LPL activity in both subcutaneous and perigonadal white adipose tissue was lower in LCHF mice than in control mice (*p* < 0.001 and *p* < 0.01, respectively) (Fig. 3a). Gene expression analysis showed similar expression levels of genes encoding inflammatory markers, hormones, and other proteins involved in lipid metabolism in subcutaneous white adipose tissue in both groups (Fig. 3b). In perigonadal white adipose tissue, the LCHF mice showed higher expression of the *Lep* gene, which encodes the hormone leptin, compared to control mice (*p* < 0.05) (Fig. 3c); the LCHF mice also showed higher expression of the gene for the macrophage-specific marker *Emr1* (*p* < 0.05). In addition, expression of the angiopoietin-like 4 gene, A*ngptl4*, which is involved in lipid metabolism by regulating LPL, was higher in LCHF mice than in control mice (*p* < 0.01).

### Liver structure, β-hydroxybutyrate analysis, and measurement of indirect calorimetry

**Fig. 4:**
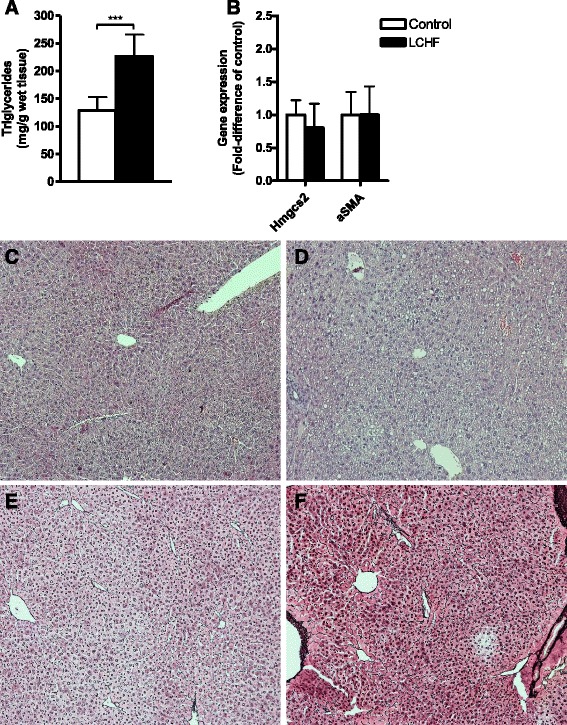
**a** Triglyceride content per gram wet liver tissue. **b** Relative gene expression in the liver normalized to that in control mice. **c, d** Representative liver sections from female C57BL/6J mice fed a low-carbohydrate high-fat (LCHF) or regular chow feed (Control) diet stained with hematoxylin-eosin. **e**, **f** Representative liver sections stained with reticulin to visualize the reticular fibers. **c**, **e** Control group. **d**, **f** LCHF group. Data are presented as mean ± SD with *n* = 7 in each group. ***p < 0.001 vs. control

The triglyceride content in the liver tissue was nearly 2-fold higher in LCHF mice than in control mice (*p* < 0.001) (Fig. 4a). In contrast, reticulin and hematoxylin-eosin staining of sections of the liver revealed no major differences in liver morphology between mice fed a control diet compared to those fed the LCHF diet. However, one mouse fed the LCHF diet showed clear signs of increased fat deposition in its hepatocytes. Moreover, analysis of the expression of *α﻿﻿SMA* that indicate stellate cell activation and hence early signs of liver fibrosis was not different in the two groups of mice, suggesting that under these conditions, the increased fat intake did not cause liver fibrosis (Fig. 4b-f).

**Fig. 5:**
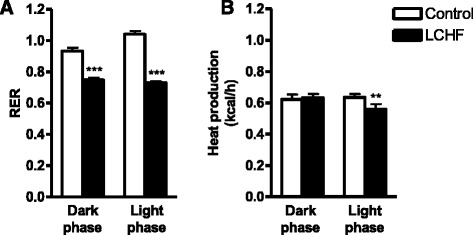
The respiratory exchange ratio (RER) and energy expenditure (kcal/h) in female C57BL/6J mice fed either a regular chow (Control) or a low-carbohydrate high-fat (LCHF) diet. **a** Mice fed the LCHF diet showed lower RERs in both the active (*dark*) phase and inactive (*light*) phase compared to control mice. **b** Energy expenditure was lower in mice fed the LCHF diet in the inactive (*light*) phase compared to control mice. No difference was seen in the active (*dark*) phase between the diet groups. Data are presented as mean ± SD with *n* = 4 in each group. **p < 0.01, ***p < 0.001 vs. control

The β-hydroxybutyrate response to the LCHF diet was highly variable within the groups. Even though there was a trend towards higher production of β-hydroxybutyrate in the LCHF group (*p* = 0.065) (Table 1), there was no difference in the two groups in the expression of the 3-hydroxy-3-methylglutaryl CoA synthase 2 gene, *Hmgcs2*, which encodes the rate-limiting mitochondrial enzyme that catalyzes the first step in β-hydroxybutyrate synthesis (Fig. 4b). Even though the LCHF group did not show significantly pronounced ketosis, indirect calorimetry showed decreased RER in both the dark and light phases (*p* < 0.001) (Fig. 5a), with RER defined as the ratio of the amount of oxygen consumed to the amount of carbon dioxide produced. In addition, energy expenditure (kcal/h) was lower in the LCHF group during the inactive (light) phase (*p* < 0.01) compared to the control group (Fig. 5b).

## Discussion

The present study showed that the weight gain of pair-fed female mice given a LCHF diet was similar to that of controls, even though their total caloric intake was reduced. Notably, 4 weeks on a LCHF diet resulted in a higher fat mass/lean mass ratio in the LCHF-fed mice. In addition, the LCHF diet impaired heart function with respect to reducing stroke volume and cardiac output, and reduced the weight of the liver and lungs relative to control mice.

The majority of diet intervention studies use *ad libitum*-fed animals. Thus, the results vary according to the amount of calories they consume, resulting in a wide spread in weight gain compared to control animals [3, 17, 18]. In this study, we wanted to evaluate the effects of the diet and to avoid the confounding effects that are the consequence of alterations in weight or overconsumption of calories [17, 18]. Therefore, mice were single housed and iso-energetically pair-fed relative to the *ad libitum* chow-fed control group. In addition, the protein content of the LCHF diet was chosen according to the Nutrient Requirements of Laboratory Animals to avoid starvation and lean mass degradation [5, 6].

After just 2 weeks on the LCHF diet, there was a 1.8-fold difference in the fat mass/lean mass ratio (*p* < 0.001) compared to controls. The changes in fat mass and lean mass were much less pronounced the two last weeks of the diet. As expected, pair feeding resulted in similar total body weight gain in both groups, but the LCHF diet resulted in higher fat mass and lower lean mass than the control diet. It has been known that dietary fats can induce obesity in rodents, independent of the total body weight gain [19, 20], and also that this could be achieved without increasing caloric intake [21, 22]. There are reports that calorie-restricted animals can preserve their fat mass [23], and 5–6 weeks on an extreme but commonly studied ketogenic diet (95 E % fat, 5 E % protein, 0 E % carbohydrates) preserves [18] or even increases [3, 17] percentage of fat mass in male mice, despite body mass reduction. In contrast to the ketogenic diet that is commonly studied in mice and that has low protein content, the LCHF diet used here has a macronutrient composition similar to what is usually found in human LCHF diets. Moreover, 20 E % protein content is well above the minimum protein requirement for normal growth, reproduction, and lactation in mice [6, 24]. In the present study, the actual protein intake was calculated to be sufficient for normal growth and development. Inspite of this, and the fact that body weight was similar to the control group, the lean mass percentage decreased substantially at the expense of fat mass following 4 weeks on the LCHF diet. In addition to the increased fat mass/lean mass ratio, we found that the weight of vital organs, such as the liver and lungs, was lower in the LCHF group. To our knowledge, no other studies have reported similar results from a protein sufficient diet. The shorter tibia length in the LCHF-fed mice could be due to reduced growth and/or changes in bone mineral content, and this needs to be further investigated [25, 26].

Lipids are the main energy source in the healthy heart, but excess lipid accumulation is associated with impaired cardiac function [27]. When carbohydrate intake is reduced, energy must be supplied by other sources, like fatty acid oxidation, that require more oxygen [27, 28]. In our current study, mice fed a LCHF diet showed higher CD36 gene expression as well as higher levels of triglycerides in the myocardium [29, 30]. Similar cardiac alterations have been reported in obese mice fed a long-term high-fat diet [30]. However, in a 2-week study on rats no change in CD36 was reported, making the duration of diet intake interesting [31]. In the present study the expression of the gene encoding natriuretic protein A (ANP) was significantly lower in the LCHF group. Increased ANP expression is traditionally used as a marker of heart failure [32, 33], but in recent years, decreases in ANP expression concomitantly with increases in the expression of its receptor, NPR-A, have been linked to metabolic dysfunction [29]. Abundant intake of dietary fat decreases ANP expression, and recent findings suggest that disturbances in the endogenous cardiac ANP/NPR-A system might induce cardiomyocyte hypertrophy [29, 32, 33]. The lower expression of ANP and the higher expression of NPR-A relative to control found in the present study might therefore reflect one way for the myocardium to counteract imminent hypertrophy [30], even though echocardiography showed that hypertrophy was not established after 4 weeks on the LCHF diet [34]. Nonetheless, we found impaired cardiac function in the LCHF-fed mice, as reflected by lower stroke volume and lower aerobic capacity compared to mice fed the control diet. The plasma glucose levels were similar in the two groups, as shown by others [34, 35]. However, in future studies it should be focus on whether the dietary intake of carbohydrates was sufficient to maintain glucose homeostasis or whether proteins contributed to the production of glucose via gluconeogenesis in the liver. The present study found no differences in insulin levels, while others report a marked reduction in insulin after 2 weeks on a similar diet [34, 35]. Increased expression of cardiac muscle ring-finger protein 1 (Murf1) and decreased expression of cardiac glucose transporter type 4 (GLUT4) might result from the organism trying to maintain normal levels of plasma glucose when dietary glucose is scarce [36]. The macronutrient composition may also be of great importance during a pathologic condition, like myocardial ischemia and ischemia-reperfusion injury. In a study by Liu et al., they demonstrated that male rats fed a 60 % fat and 10 % carbohydrate diet for 2 weeks display an increased myocardial infarct size compared to rats fed a standard rodent diet, even though no differences were seen during baseline conditions (*i.e* before induction of myocardial infarction) [31]. The authors also showed that cardiac GLUT1 gene expression was significantly downregulated, which suggests less glucose available as energy substrate during anaerobic conditions. Impaired recovery of ischemic myocardial injury, as well as attenuation of mitochondrial biogenesis has been found in obese rats fed a low-carbohydrate high-fat diet when subjected to an ischemia/reperfusion injury, or coronary artery ligation [34, 37]. Variances in macronutrient composition could therefore be well tolerated by healthy hearts, but during hypoxic conditions inadequate supply of carbohydrates may be detrimental.

Liver histology did not show increased fibrosis or steatosis, even though the hepatic triglyceride content had increased. The lower liver weight seen in the LCHF group could be due to overall degradation of the tissues that facilitate gluconeogenesis with amino acids, and hypoplasia would not affect the morphology per se.

Under standard housing temperature conditions (21–23 °C), mice compensate for increased thermogenesis by increasing their energy intake [38]. In the present study, we found that the LCHF group had significantly lower heat production as measured by indirect calorimetry during the inactive (light) phase. If the observed reduction in lean mass was due to insufficient energy supply, lower physical activity or tissue degradation in response to changes in the metabolic state merit further investigation. Still, this could partly explain why mice fed a LCHF diet could maintain their body weight in spite of a somewhat lower total energy intake. In male mice, high-fat diets result in an increased prevalence of low-grade inflammation in adipose tissue as well as increased plasma glucose, insulin, and triglyceride levels; the exception is that when a ketogenic state is reached, the insulin level decreases [3, 8, 17]. In this short study, mice were fed a LCHF diet for just 4 weeks, and this did not alter the fasting glucose, total triglyceride, insulin, or non-esterified fatty acid levels. These results are in line with the plasma findings in female mice fed a low-carbohydrate diet for 14 weeks in a study by Pettersson et al. [8].

Adipose tissues secrete a vast number of cytokines, chemokines, hormones, and other factors that regulate metabolic pathways [39]. Many of these so-called adipokines show altered expression patterns in obesity. The LCHF diet did not alter the expression of genes encoding adipocyte-derived hormones (*lep* and *adipoq*) or monocyte/macrophage-related genes (*Tnf*, *Ccl2*, and *Emr1*) in subcutaneous adipose tissue. In perigonadal adipose tissue, however, the LCHF diet induced a significant increase in leptin and F4/80, *Emr1*. Leptin plays a key role in the regulation of energy intake and energy expenditure, and its circulating concentration is proportional to body fat mass [40]. Leptin levels increase in states of energy excess, such as in obesity and overconsumption of calories, but the effect of leptin in the central nervous system is blunted due to leptin resistance. It is reasonable to assume that the higher expression of the leptin gene that we observed in the perigonadal adipose tissue of LCHF mice (and the similar trend in subcutaneous fat) was caused by the increased fat mass in these animals. It is likely that the increased leptin expression leads to higher circulatory levels of leptin. Thus, it is plausible that elevated adipose leptin production is at least partly responsible for the reduced food intake at the end of the study by mice fed a LCHF diet.

Elevated leptin levels might also enhance energy expenditure through sympathetic activation. In rodents, leptin stimulates thermogenesis [41, 42]. The indirect calorimetry measurements in our study revealed that energy expenditure did not differ between the diet groups in the active (dark) phase, but interestingly, heat production was actually lower in mice fed a LCHF diet in the inactive (light) phase. This could explain why mice fed the LCHF diet could maintain their body weight despite somewhat lower total energy intake. About a decade ago, it was reported that adipose tissue in obese individuals is characterized by macrophage infiltration and that the macrophages are an important source of inflammation [43]. Notably, the number of macrophages in rodent and human adipose tissue increases in proportion to the adipose tissue mass. It is therefore thought that an increase in adipose tissue macrophage density has a pronounced impact on adipose function, as macrophage-secreted factors evoke an inflammatory response in adipocytes along with increased lipolysis and reduced glucose uptake [44]. Although the LCHF diet in the current study had pronounced effects on the amount of adipose tissue, the expression levels of monocyte/macrophage-related genes did not differ in the two diet groups except that the gene expression of the macrophage marker F4/80 *Emr1* in perigonadal fat was very slightly, although significantly, higher in LCHF animals. These data suggest that female mice fed a LCHF diet for 4 weeks do not activate the signaling pathways and subsequently the expression of pro-inflammatory cytokines in their adipose tissue depots. This may be explained by sex differences. In the literature, there are data suggesting that female mice are protected from some of the metabolic derangements induced by high-fat diets. For example, Pettersson and colleagues reported that despite similar weight gain in male and female mice following 14 weeks on a high-fat diet (60 E % fat), male but not female mice developed visceral inflammation, glucose intolerance, insulin resistance, and hyperinsulinemia [8]. Interestingly, and in contrast to observations in male mice, the female mice maintained an anti-inflammatory environment in the intra-abdominal adipose tissue. In the present study we used female mice because they are more resistant to diet-induced metabolic changes [7]. The female sex hormones are thought to be protective against metabolic syndrome and low-grade inflammation [8, 45, 46]. In humans, there are fewer incidents of obesity-related metabolic disorders prior to menopause in women [47]. However, some obesity-related metabolic alterations are independent of sex, such as changes in ANP and NPR-A levels [29]. Thus, future studies are warranted to investigate possible sex-specific effects of a LCHF diet.

## Conclusion

The present data demonstrated that female mice that were iso-energetically pair-fed a LCHF diet (75 E % fat, 20 E % protein, 5 E % carbohydrates) showed a higher fat mass/lean mass ratio than control mice after just 2 weeks. After 4 weeks, they showed lower liver and lung weights, and there was a similar trend for the heart. Importantly, their cardiac function and maximum aerobic capacity was impaired after 4 weeks on the LCHF diet. It is not known whether any of these undesirable effects occur in humans that choose to eat low-carbohydrate diets; however, the current data suggest that the effects of low-carbohydrate diets on cardiac function should be carefully evaluated in humans.

## Abbreviations

LCHF: Low-carbohydrate high-fat diet
NEFA: Non-esterified fatty acids
RER: Respiratory exchange ratio
ANP: Atrial natriuretic peptide
NPR-A: Atrial natriuretic peptide receptor
GLUT1/4: Glucose transporter ¼
LPL: Lipoprotein lipase
